# Advances in Atomic Force Microscopy: Weakly Perturbative Imaging of the Interfacial Water

**DOI:** 10.3389/fchem.2019.00626

**Published:** 2019-09-12

**Authors:** Duanyun Cao, Yizhi Song, Jinbo Peng, Runze Ma, Jing Guo, Ji Chen, Xinzheng Li, Ying Jiang, Enge Wang, Limei Xu

**Affiliations:** ^1^International Center for Quantum Materials, School of Physics, Peking University, Beijing, China; ^2^Institute of Experimental and Applied Physics, University of Regensburg, Regensburg, Germany; ^3^College of Chemistry, Beijing Normal University, Beijing, China; ^4^School of Physics, Peking University, Beijing, China; ^5^Collaborative Innovation Center of Quantum Matter, Beijing, China; ^6^CAS Center for Excellence in Topological Quantum Computation, University of Chinese Academy of Sciences, Beijing, China; ^7^Ceramics Division, Songshan Lake Materials Lab, Institute of Physics, Chinese Academy of Sciences, Guangdong, China; ^8^School of Physics, Liaoning University, Shenyang, China

**Keywords:** interfacial water, atomic force microscopy, quadrupole-like CO-terminated tip, ultrahigh-resolution, intrinsic structural determination

## Abstract

The structure and dynamics of interfacial water, determined by the water-interface interactions, are important for a wide range of applied fields and natural processes, such as water diffusion (Kim et al., 2013), electrochemistry (Markovic, 2013), heterogeneous catalysis (Over et al., 2000), and lubrication (Zilibotti et al., 2013). The precise understanding of water-interface interactions largely relies on the development of atomic-scale experimental techniques (Guo et al., 2014) and computational methods (Hapala et al., 2014b). Scanning probe microscopy has been extensively applied to probe interfacial water in many interdisciplinary fields (Ichii et al., 2012; Shiotari and Sugimoto, 2017; Peng et al., 2018a). In this perspective, we review the recent progress in the noncontact atomic force microscopy (nc-AFM) imaging and AFM simulation techniques and discuss how the newly developed techniques are applied to study the properties of interfacial water. The nc-AFM with the quadrupole-like CO-terminated tip can achieve ultrahigh-resolution imaging of the interfacial water on different surfaces, trace the reconstruction of H-bonding network and determine the intrinsic structures of the weakly bonded water clusters and even their metastable states. In the end, we present an outlook on the directions of future AFM studies of interfacial water as well as the challenges faced by this field.

## Introduction

Water-interface interactions are of vital importance in both fundamental science (Xu et al., 2010) and application fields (Markovic, 2013). The structure of interfacial water could be easily influenced by the surrounding environment and interfacial heterogeneity, due to the delicate competition between water-surface and water-water interactions. Therefore, to understand the unusual properties of interfacial water, atomic-scale structural imaging is critical. Furthermore, limited by spatial resolution, using conventional spectroscopic and diffraction techniques [e.g., sum-frequency generation (Shen and Ostroverkhov, 2006), X-ray diffraction (Nakamura and Ito, 2005), and nuclear magnetic resonance (Matubayasi et al., 1997)] to investigate the interfacial water would easily blur the fine details of water-interface interaction. So far, a variety of surfaces and nanostructured systems could be characterized and manipulated at the atomic-scale by scanning probe microscopy (SPM), involving scanning tunneling microscopy (STM) (Guo et al., 2014) and atomic force microscopy (AFM) (Xu et al., 2010). Thus, SPM is an effective technique to detect the microstructure and dynamics of interfacial water. STM measures tunneling current between the probe and metallic substrates, which limits its applicability on non-conductive surfaces (Morita and Sugawara, 2001); while AFM probes tip-sample atomic forces, making it suitable for a wider range of interfacial water systems (Thürmer and Nie, 2013). Furthermore, due to the intricate relation between the AFM signal and the measured structure, AFM modeling plays a pivotal role in interpreting the experimental results. This paper reviews recent progresses in AFM imaging and simulation techniques in Section “Recent advances in AFM imaging and AFM simulation methods”, and discusses how the newly developed AFM are utilized to detect the interfacial water with molecular or even atomic scale resolution in Section “The ultrahigh-resolution imaging of interfacial water”. Finally, some new techniques beyond current applications to the study of interfacial water are conceived in Section “Perspective”, and a summary is given in Section “Conclusion”.

## Recent Advances in AFM Imaging and AFM Simulation Methods

### High-Resolution Imaging With Functionalized Tip

The submolecular resolution imaging of molecular structures on surfaces fully deserves one of the most outstanding achievements of AFM in recent years. Albrecht and coworkers introduced frequency modulation (FM) detection using oscillating cantilever (Albrecht et al., 1991). As the probe is not in contact with the surface directly, this technique is widely known as the nc-AFM (Giessibl, 2003). After the first achievement of the stable and real atomic resolution of the Si(111) surface (Giessibl, 1995), this technique has been developed rapidly (Morita and Sugawara, 2001). Later, using the qPlus based nc-AFM, the chemical structure of a single pentacene molecule has been resolved with a CO-tip by probing the short-range Pauli repulsion force (Gross et al., 2009). The apparent distortion and sharpening of bonds in high-resolution AFM images were attributed to the relaxation of the functionalized tip apex, namely the incline of CO under the action of external forces (Gross et al., 2012). In addition to CO functionalized tip, various defined functionalized AFM tips, such as Cl (Gross et al., 2009), Br (Mohn et al., 2013), Xe (Mohn et al., 2013), and NO (Mohn et al., 2013) tips, can be constructed and used to visualize nanostructures on surfaces. For instance, atomic contrast can be achieved with the Xe-tip (Schuler et al., 2013), but there are no strong distortions or bonds sharpening as that with CO-tip, possibly due to different charge distribution at the tip apex (Peng et al., 2018b). Tip functionalization has been applied to various systems, such as organic molecules (Jarvis, 2015), metal clusters (Emmrich et al., 2015), and 2D materials (Barja et al., 2016). In addition to superior resolving and distinguishing the chemical structure, nc-AFM has also been applied to probe the intermolecular interaction (Gross et al., 2009), bond order (Gross et al., 2012), the intrinsic structure of natural product (Gross et al., 2010) chemical reaction products (de Oteyza et al., 2013), charge distribution (Mohn et al., 2012) and so on.

### Simulation of High-Resolution Imaging With Functionalized Tip

The intricate relation between the AFM signal and the measured structure, for instance sharp ridges observed by AFM between atoms without the real chemical bond, signify that the mechanism of high-resolution imaging is worth investigating. A large number of different methods have been used to simulate and interpret the AFM images (Giessibl et al., 2000; Caciuc et al., 2006; Reischl et al., 2016). The probe-particle model established by Hapala et al. (2014a) is capable of reproducing multiple features in the experimental image and was widely used to simulate the high-resolution AFM imaging. To simulate tip apex relaxation, their model describes functionalized tip as a combination of the tip base (the outermost atom of the metal tip) and the probe particle attached to it. Based on this simplification, they established a force-field model considering Lennard Jones forces and showed that the sharp lines in AFM images actually represent ridges connecting two minima on the potential energy landscape caused by adjacent atoms.

Later, they found that AFM images could provide information for charge distribution within molecules on the surface and extended their model by considering electrostatic forces acting on the decorated tip in the surface Hartree potential obtained from the Density Functional Theory calculation (Hapala et al., 2014b). They showed that electrostatic interaction dominates the shape of the tip-sample potential energy at large tip-sample distances, and the frustrated translation of CO is susceptible to the local curvature of sample potential. Later, Peng and Hapala et al. found that the simulated AFM images of interfacial water using a quadrupole-tip are in good agreement with the experimental result using CO-tip (Peng et al., 2018b), which is discussed in the next section in detail. Besides, the dipole of the metal tip-base (Schulz et al., 2018), considering the Smoluchowski effect, is an option to be applied if needed. Moreover, they upgraded the model by taking CO as two probe particles (Di Giovannantonio et al., 2018).

## The Ultrahigh-Resolution Imaging of Interfacial Water

AFM has been widely used in numerous interdisciplinary studies of interfacial water (Su et al., 2010), however, the tip intrusion into the H-bonding networks has been tricky to deal with. Generally, at small tip-sample distances, atomic resolution is achieved with the prominent relaxation of tip apex due to the dominant strong tip-sample Pauli repulsion (Gross et al., 2009). Whereas, for water structures, H-bond is so weak that it could be easily destroyed by the disturbance in this way (Shiotari and Sugimoto, 2017). At large tip-sample distances, where just long-range electrostatic and van der Waals forces are measurable, the resolution of weakly polarized molecule is usually very low. Fortunately, water molecules have strong dipole moments, thus high-resolution AFM imaging of water can be obtained by using suitable charged tip apex (Ellner et al., 2016). In this section, we discuss AFM studies of water networks (Shiotari and Sugimoto, 2017), nanoclusters (Peng et al., 2018b), and ion hydrates (Peng et al., 2018a).

### Imaging of Water Networks

The wetting process on metal surfaces is closely related to the local defects existing in water layers growing on the surface, which are hardly resolved by spectroscopic methods. Using AFM with a CO-tip, Shiotari and Sugimoto demonstrated ultrahigh-resolution imaging of water monolayers including 1D water chains, local defects in the water chains and water-hydroxyl network on Cu(110) surface (Shiotari and Sugimoto, 2017). In the AFM imaging, hydrogen atoms contribute slightly, while the oxygen atoms are predominant, displaying water networks containing edges and local defects composed of pentagonal and hexagonal rings. The AFM imaging provides valuable information that are not achievable using other techniques. For instance, atomic structures of local defects in water networks can be well-distinguished in AFM images, while they are unclear in STM images; the cluster composed of a hexagonal ring surrounded by four pentagonal rings are revealed with AFM, but were imaged as tetraphyllous-shaped protrusions with STM. In the observation of H_2_O-OH mixed network, they found that although H atoms are invisible in the AFM images, the O-O distance correlated highly with the strength of H-bonds can be sensitively detected, thus H-bonds can be detected by AFM. Furthermore, they demonstrated that AFM imaging can trace the reconstruction of H-bonding network in real time, although it can be rearranged readily and is more flexible than the covalently bonded organic molecules. These findings further demonstrated that AFM is practical to characterize atomic structures of weakly bonded molecular structures, and its application to water systems leads to major breakthroughs in the study of water-solid interfaces.

### Structural Determination of Weakly Bonded Water Clusters and Even Their Metastable States

**Figure 1:**
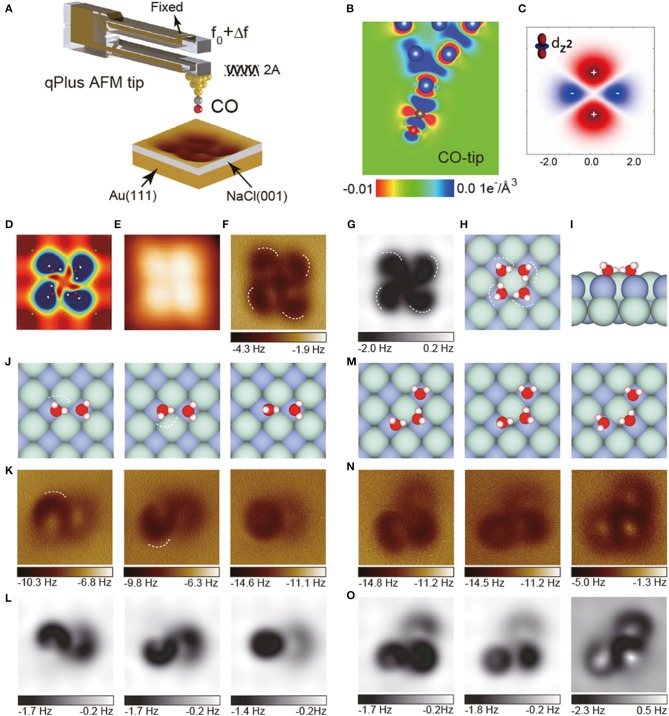
AFM images of water clusters with a CO-terminated tip. **(A)** Schematic of a qPlus-based nc-AFM with a CO-tip. The cantilever oscillates at an amplitude of A and the tip-sample force-induced frequency shift of the cantilever from its natural resonance frequency (f_0_) is Δf. **(B)** Charge distribution of the CO-tip from DFT calculations. **(C)** xz-cut of the charge distribution of quadrupole ($d_{z^{2}}$) tip model. **(D)** Calculated electrostatic potential map of the water tetramers in a plane 60 pm above the outermost H atom. **(E)** Constant-current STM images acquired at (100 mV, 20 pA). **(F)** Experimental Δf images recorded at the tip heights of 100 pm. The tip height is referenced to the STM set point on the NaCl surface (100 mV, 50 pA). **(G)** Simulated AFM images of water tetramer. **(H,I)** Top and side view of the water tetramer adsorbed on the NaCl(001) surface, respectively. **(J–L)** Geometric structures, experimental and simulated Δf images of weakly bonded water dimers, respectively. The crooked depressions in the AFM images are highlighted by dashed lines in **(K)**. **(M–O)** Geometric structures, experimental and simulated Δf images of weakly bonded water trimers, respectively. H, O, Cl, and Na atoms in the atomic models are denoted as white, red, cyan, and purple spheres, respectively. The oscillation amplitude is 100 pm. All the simulations were done with a quadrupole ($d_{z^{2}}$) tip (*k* = 0.5 N m^−1^, Q = −0.2 e). The size of the images is 1.2 × 1.2 nm. Adapted with permission from Peng et al., (2018b).

In 2018, by detecting the high-order electrostatic force utilizing AFM with a CO-tip, Peng et al. presented weakly perturbative atomic resolution imaging of water clusters on Au(111)-supported NaCl(001) surface (Peng et al., 2018b). In the experimental study of water tetramer (Figure 1A), the STM image shows only basal steady features (Figure 1E), while AFM image shows obvious internal features (Figure 1F) which is similar to the calculated electrostatic potential distribution (Figure 1D). The charge density difference of the CO-tip (Figure 1B) displays that the tip peak resembles quadrupole (Figure 1C). Theoretical simulation based on the probe-particle model with a quadrupole-tip (Figure 1G) reproduced the experimental AFM image perfectly, revealing that the ultrahigh-resolution in the AFM image is due to the weak high-order electrostatic force between water molecules and the quadrupole-like CO-tip. The AFM image displays the depression features, which are directly correlated with H (Figures 1F–H, white dashed lines), allowing us to precisely identify the H-bonds and detailed structures of water clusters.

Surprisingly, although the water-tip interaction is weak at large tip heights, the structures and even metastable states of weakly bonded water clusters, such as water dimers and trimers, can be accurately determined almost without intrusion with AFM. Slight differences in the O-H incline of water dimers can be easily distinguished by AFM (Figures 1J,K, white dashed lines). Moreover, although being more unstable and having multiple metastable states, water trimers (Figure 1M) can still be imaged with ultrahigh-resolution (Figure 1N). Combined with simulations (Figure 1O), their atomic structures can be determined without ambiguity. The differences in adsorption energies of different metastable states of water trimers are very small (<47 meV), allowing the structures to fluctuate rapidly among different states under external perturbation. The ability to identify them suggests that the probe is almost non-invasive.

### Determination of the Particle Charge State and the Ion Hydrate Structure

**Figure 2:**
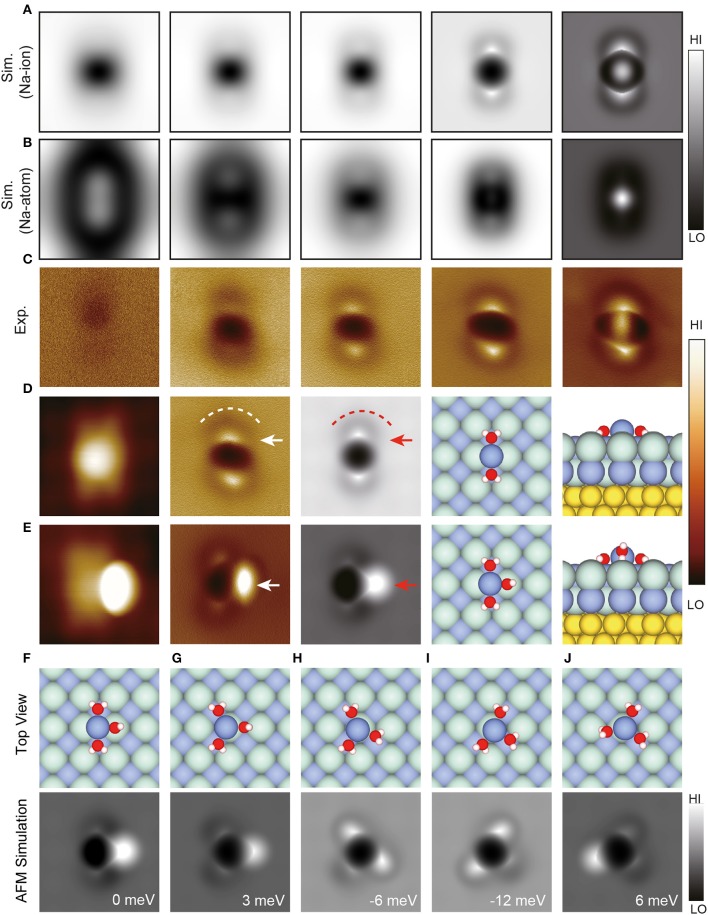
Geometries and high-resolution STM/AFM images of Na^+^ hydrates. **(A,B)** Simulated AFM images of Na^+^•2H_2_O and Na•2H_2_O, respectively. The tip heights of the simulated AFM images in **(A,B)** from left to right are 11 Å (13.05 Å), 10.05 Å (12.05 Å), 9.35 Å (10.65 Å), 8.05 Å (9.15 Å), and 7.5 Å (8.7 Å), respectively. These images were chosen to show the typical feature change as the tip height decreased. **(C)** Experimental Δf images of Na^+^•2D_2_O recorded at the tip heights of 220, 150, 100, 50, −10 pm, respectively. **(D,E)** The STM/AFM images (acquired with a CO-tip), AFM simulations and atomic models (left: top view; right: side view) of Na^+^•nD_2_O clusters (*n* = 2–3), respectively. **(F–J)** Atomic models (top view) and simulated AFM images of different types of Na^+^•3H_2_O. The relative energy of different Na^+^•3H_2_O with respect to the total energy of the structure in **(E)** was labeled at the bottom right of simulated AFM images. The white (red) arrows in **(D)** denote bright protrusions, and the white (red) dashed lines highlight the crooked depressions in the AFM images (simulations). The white (red) arrow in **(E)** denotes the standing water in AFM image (simulation). Set point of STM images **(D,E)**: *V* = 150 mV and *I* = 30 pA, *V* = 100 mV, and *I* = 30 pA, respectively. The tip height of experimental AFM images is referenced to the STM set point on the NaCl surface (100 mV, 50 pA). The tip height in simulations is defined as the vertical distance between the apex atom of the metal tip and the Na^+^ ion in Na^+^ hydrates. H, O, Cl, Na and Au atoms in the atomic models are denoted as white, red, cyan, purple, and yellow spheres, respectively. All the AFM oscillation amplitudes of experimental and simulated images are 100 pm. All the AFM simulations were done with a quadrupole ($d_{z^{2}}$) tip (*k* = 0.75 N/m, Q = −0.2 e). The size of the images: 1.5 ×1.5 nm. Adapted with permission from Peng et al., (2018a).

In addition to water clusters described above, Peng et al. also studied the Na^+^ ion hydrate, in which Na was positively charged and its charge state was confirmed by comparing the experimental and simulated images (Peng et al., 2018a). For Na^+^•2H_2_O, the simulated AFM images (Figure 2A) are in good agreement with experimental results (Figure 2C). In contrast, for Na•2H_2_O, the simulated AFM images (Figure 2B) show significant deviation from the experimental results. Na^+^ is shown as a dark feature in AFM images, mainly due to its electrostatic attraction with the CO-tip. Water molecules appear as a bright feature (Figure 2D, white arrow) surrounded by a dark ring (Figure 2D, white dashed line), on account of the charge state of O and D, respectively.

For Na^+^•3H_2_O, it is difficult to predict the most stable structure (Figures 2F–J) according to calculations alone due to the small energy difference (<20 meV) of different states. Thanks to the ultrahigh-resolution experimental and simulated AFM images, the perfect agreement between the simulated AFM image in Figure 2F and the experimental AFM image in Figure 2E can be distinguish, revealing that Figure 2F is the accurate structure of Na^+^•3H_2_O. Furthermore, Na^+^•3H_2_O was found to diffuse orders of magnitude faster than other hydrates, arising from its multiple metastable states, in which water molecules can rotate collectively around Na^+^ with a minimal energy barrier. It is promising that this technique will be extended to other hydration systems, opening the door to further study of various hydration processes with atomic resolution.

## Perspective

### AFM Imaging of Ice Formation

Ice formation on exposed surfaces play critical roles in an incredibly broad spectrum of atmospheric science (Kiselev et al., 2017), materials science (Parent and Ilinca, 2011), biology (Graether et al., 2000), and planetary science (Head et al., 2003). However, the microscopic mechanisms of ice nucleation remain unclear (Gerrard et al., 2019). Experimental investigation of the ice formation with atomic precision remains to be a grand challenge so far, due to the short lifetimes of the intermediate structures and fragileness of the ice edges (Lupi et al., 2014). Particularly, determining the transition from single layer to multilayer adsorption is a great challenge, because the structure and surface energy of the first water layer are generally quite different from that of a bulk ice film and the evolution from the 2D to 3D ice growth is complex. Although both SPM and spectroscopic techniques have been used to study multilayer growth, atomic scale characterization of H-bonds in ice structure and understanding of the ice growth process are still lacking (Thürmer and Nie, 2013; Gerrard et al., 2019). Development of advanced SPM techniques, particularly the weakly perturbative AFM imaging, is likely to play a considerable role in this area.

### AFM Imaging in Liquids

The high-resolution AFM studies in liquid were sparse at first, which was hindered by the reduced Q-factor of the cantilever (Kobayashi et al., 2002) and many other reasons such as the high mobility of liquid particles. The first atomic resolution AFM imaging in liquid was achieved by Fukuma et al. with the reduced frequency noise and small cantilever oscillation amplitude in 2005 (Fukuma et al., 2005). Such high-resolution imaging in water with minimal deflection noise density (Fukuma and Jarvis, 2006) opened up the possibility of submolecular-scale imaging of liquid/solid interface as well as materials in liquid (Fukuma et al., 2005; Tracey et al., 2016). Lately, using stiff qPlus sensors with small amplitude was found to be able to obtain high Q-factors and image soft biological samples in liquid (Pürckhauer et al., 2018). The subsequent improvement of AFM in force sensitivity, operation speed and other basic performances will significantly broaden the application of AFM in liquid-environment. Meanwhile, the development of AFM based techniques of measuring surface properties (e.g., potential, charge distribution, viscoelasticity, hydrophilicity, and chemical sensitivity) will help to understand the mechanism of AFM contrast and further study various properties of interfacial water. The development of these instruments and their applications have already begun, for example the high-resolution surface potential which reflects the charge distribution can be measured using Kelvin probe force microscope (Nonnenmacher et al., 1991), and AFM is a promising tool to investigate interfacial water in liquid environment.

### Structural Dynamics Studied With High-Speed AFM (HS-AFM)

Although AFM experiments can provide extensive information, its relatively long data acquisition time makes it difficult to track numerous structural transitions and dynamic processes at the nano- to microseconds time scales (Muller, 2008). As a result of the rapid development of various techniques and devices in recent years, the HS-AFM demonstrated its innovative capability by visualizing several nanostructural dynamic processes occurring in biological protein systems and large samples (including isolated intracellular organelles, living cells, and DNA nanostructures (Uchihashi et al., 2011; Ando et al., 2013, 2014; Suzuki et al., 2015; Sutter et al., 2016), such as structural transitions, mechanical actions, self-assembly processes, dynamic interactions with partners and so on. These studies made important discoveries and provided significant insights that are not accessible by other approaches. In contrast, applications of HS-AFM to the interfacial water systems were limited since its resolution is not high enough to distinguish different water molecules (Liao et al., 2018). By improving AFM control systems for its better speed performance and functionality, HS-AFM will have a considerable impact on the structural dynamics studies of interfacial water, such as the diffusion of water clusters and ice growth on different surfaces.

### Manipulation With AFM at Higher Temperature

Manipulation of atoms and molecules on surfaces can provide a means to deepen understanding of basic chemical and physical processes at surfaces. AFM manipulation has become an attractive research field in recent years since it can be conducted at both cryogenic temperature (Ternes et al., 2008) and room temperature (Sugimoto et al., 2007), and the involved driving forces which provide the specific signature of a certain mechanical manipulation process can be measured. During the manipulation, the interaction between the tip and surface is vital to induce the reduction of local energy barrier, which can be applied for a variety of purposes such as facilitating chemical reactions (Schuler et al., 2016), fabrication of atomic-sized materials (Sugimoto et al., 2014) and atomic-scale logic devices (Loth et al., 2012). However, the stable assembling of clusters and the precise determination of cluster size at higher temperature remain challenging (Ming et al., 2011). Moreover, the manipulation of multi-element nanoclusters at room temperature has not been achieved, let alone that of water molecular nanoclusters which can provide a unique opportunity of studying water-surface and water-water interaction under a well-defined environment closely related to daily issues. Further progresses in AFM are expected for the broad technical potential of this assembly.

### Modeling of the AFM Imaging

Atomic-scale insight into a variety of researches [e.g., friction (Sheehan and Lieber, 2017), crystal engineering (Chow et al., 2012), self-assembly (Schreiber et al., 2013), catalytic reactions (Over et al., 2000), electron transport (Wang et al., 2010) and reactions in electrochemistry (Markovic, 2013)], have been provided by many pioneering experimental AFM studies. The intricate relation between the AFM signal and the measured structure requires the development of theoretical modeling which really provides eligible simulation of experiment. Simulations of AFM experiments in ultrahigh vacuum (UHV) environment were well-developed with many modeling approaches. However, the modeling to characterize the AFM imaging of the solid-liquid interfaces, especially the measurements outside vacuum conditions or those dynamic processes (Chow et al., 2012; Schreiber et al., 2013), becomes much harder and lags far behind experiments.

At present, it is really hard to interpret many revolutionary experiments (Kada et al., 2008; Müller and Dufrêne, 2008), in which AFM suffers from the invasive imaging and potential damage to samples, particularly in water systems where the molecules are usually just physically adsorbed on the surface by weak van der Waals interactions. In addition, the forces on the tip would further cause its relaxation, indicating the necessary of molecular dynamics (MD) techniques (Allen and Tildesley, 2017). The timescales required to equilibrate the system usually prevent quantum mechanical methods from modeling these experiments (Holmberg et al., 2014), leading to the demand of the precise classical approach (Watkins and Shluger, 2010; Reischl et al., 2013) which is nontrivial as the complexity of materials and environments increases. However, these applications are critical to keeping AFM at the front of next-generation characterization technologies, so AFM modeling needs to be well developed and will continue to play a pivotal role.

## Conclusion

In summary, we discuss the recent advances in AFM and how the newly developed techniques are applied to the study of interfacial water. Due to the intricate relation between the AFM signal and the measured structure, AFM modeling plays a pivotal role in interpreting the experiments. AFM with the CO-tip can achieve ultrahigh-resolution imaging of the interfacial water, trace the reconstruction of H-bonds and determine the intrinsic structures of the weakly bonded water clusters and even their metastable states. We present the prospect of future AFM studies of more complex and realistic interfacial water-related systems. It is beyond doubt that the continuous improvement of AFM imaging and AFM modeling in the coming decades will bring about more comprehensive understanding of the structural, mechanical, dynamic and functional heterogeneity of intricate interfacial water systems and enable solutions to the outstanding issues.

## Author Contributions

DC, YS, and LX wrote the manuscript. The manuscript reflects the contributions of all authors.

### Conflict of Interest Statement

The authors declare that the research was conducted in the absence of any commercial or financial relationships that could be construed as a potential conflict of interest.
